# Spillover effects of competition outcome on future risky cooperation

**DOI:** 10.1038/s41598-023-32523-6

**Published:** 2023-04-04

**Authors:** Yansong Li, Zhenliang Liu, Yuqian Wang, Edmund Derrington, Frédéric Moisan, Jean-Claude Dreher

**Affiliations:** 1 Reward, Competition, and Social Neuroscience Lab, Department of Psychology, School of Social and Behavioral Sciences, Nanjing University, Xianlin Avenue 163, Nanjing, 210023 China; 2 Department of Radiology, The Affiliated Drum Tower Hospital of Nanjing University Medical School, Nanjing, China; 3 Institute for Brain Sciences, Nanjing University, Nanjing, China; 4 EM-Lyon and GATE CNRS, Ecully, France; 5 Institute of Cognitive Science Marc Jeannerod, CNRS, Lyon, France; 6 Nanjing University, Nanjing, China

**Keywords:** Psychology, Human behaviour

## Abstract

There is growing evidence that risky cooperation is regulated by the experience of previous interactions with others. However, it is unclear how the evaluation of outcomes from competitive interactions can affect individuals’ subsequent cooperative behavior. To address this issue, we examined how participants cooperated with a partner having just competed with them. While competing, participants (N = 164) were randomly assigned to receive one of four types of outcome feedback regarding their performance (victory vs. defeat vs. uncertain vs. no competition (control)). We found that both the experience of defeats and of uncertainty as competitive outcomes exerted a negative impact on the extent to which participants then engaged in cooperative behavior with their recent opponents. This only occurred when such subsequent cooperative behavior involved a high potential for incurring personal costs but not when there was no risk of incurring personal costs and a positive return. Finally, mediation analysis revealed that the effect of defeat was mediated by participants’ level of interpersonal trust and the extent to which participants were willing to cooperate, while the effect of the uncertain competitive outcome was mediated only by the extent to which participants were willing to cooperate. These findings offer novel insights into how risky cooperation is modulated by previous competition.

## Introduction

“We have no eternal allies, and we have no perpetual enemies. Our interests are eternal and perpetual…….”

——Lord Palmerston (Henry John Temple)

Cooperation is a form of social interaction widely observed in humans and non-human social species^1,2^. Since both personal and mutual benefits are usually dependent on one’s own actions as well as the unknown actions of others, most forms of cooperative behavior entail an element of risk and as a consequence, can be described as risky cooperation^3^. Over the past decades, substantial efforts have been devoted to determining which variables impact risky cooperation using economic games in humans^2–4^. Social factors such as communication and group identity have previously been shown to influence cooperation^5–7^.

One potentially critical social factor has remained largely unstudied, because the vast majority of existing research has primarily focused on the impact of cooperative decisions between individuals that are 'anonymous'. This approach may fail to fully capture the essence of cooperative interactions since, in daily life, interactions between individuals are generally relationally-dependent^8^. Thus, individuals’ cooperative behavior towards others is likely to vary as a consequence of who is involved and the nature of their previous interactions. In recent years, there have been increased efforts to examine the role that such relational variables may play in explaining variations in risky cooperation^2,3,9–12^. For instance, earlier studies found that individuals who are wealthier or who can profit more from collectively shared public goods are more cooperative^13–15^. One study found that disproportionate power to impose sanctions also has a positive impact on risky cooperation^16^, although a recent study yielded contradictory results using a different type of economic game^17^. These findings illustrate the constructive role of some forms of social relations across individuals in favoring risky cooperation.

Recently there has been an increasing interest in examining how exposure to competitive interactions impacts individuals’ risky cooperation. A body of literature at the interface of psychology and behavioral economics has consistently reported that individuals embedded in a competitive setting showed higher levels of cooperation as reflected by their increased contribution to a public good^18–23^ or a more homogenous distribution of resources between them and opponents (compared to a random distribution)^24^. However, in the context of simultaneous decision-making in competitive and cooperative games, existing literature has revealed that individuals’ behavior in cooperative games was not affected by their participation in competitive games^25,26^. Despite these promising findings, the extent to which risky cooperation might be influenced by outcome-based evaluations of previous competitive experiences has been largely overlooked by previous research. This is an important issue because there are many real-world situations in which two identifiable people or entities (such as companies or even countries) compete first and then engage in risky cooperation. For example, in business, two companies may compete in the marketplace but later collaborate on a project or joint venture. In sports, athletes may compete against each other in a game or tournament but later work together as teammates on a national or international team. In politics, two countries may engage in diplomatic competition but later cooperate on international issues such as trade or security. As an emerging area of concern, a recent study provides insight to advance work within this domain^27^. In this study, participants took part in a two-stage, online experiment. In the first stage, participants, randomly assigned to one of four treatment groups, competed in a simple task that determined their earnings. The incentive scheme differed across four groups: the lottery group, the competition without outcome feedback group, the competition with outcome feedback group, and a control group that underwent no pretreatment^27^. In the second stage, all participants engaged in a standard public good game (PGG) with a different set of participants, not the same individuals they had interacted with in the first stage. The major findings of this study revealed that the competition without outcome feedback group contributed less in the PGG. Furthermore, in the group that received feedback, the losers contributed less to the public good than the winners in the PGG. However, it remains to be determined how competition outcome affects contestants’ subsequent risky cooperative behavior with their previous opponents. Given that an increase in marginal return to an individual from contributions to the public good tends to enhance contributions^28–30^, it also remains unknown how variations in the marginal per-capita return (MPCR) for the PGG might modulate any behavioral change in contribution resulting from the outcome of the competitive experience and what the underlying psychological mechanism might be.

**Figure 1:**
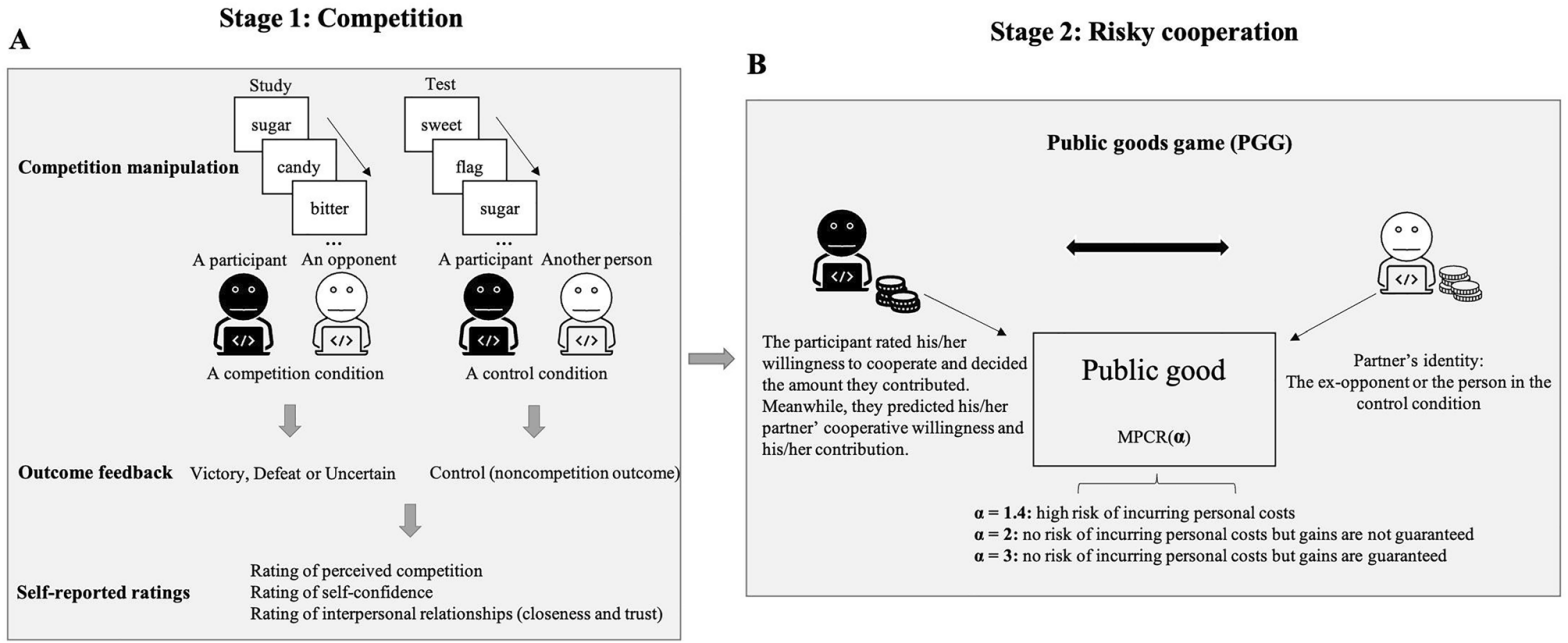
Experimental task structure. (**A**) The competition with outcome feedback manipulation is represented. In a between-participants design, participants were randomly assigned to receive one of four types of outcome feedback (victory vs. defeat vs. uncertain competitive outcome vs. no competition (control)). We used a standard version of the Deese–Roediger–McDermott (DRM) paradigm as the competitive task. At the end of the competition, participants were immediately given information about their performance. Specifically, they received one of three types of outcome feedback (victory vs. defeat vs. uncertain competitive outcome). After receiving outcome feedback, participants were asked to provide a rating on a nine-point Likert scale of their perceived competition (the intensity they felt about the competition) (1 = not at all, 9 = extremely), self-confidence (1 = not at all confident, 9 = completely confident), the degree of closeness that they felt toward their opponents (1 = not at all close, 9 = completely close) and the level of trust that they felt toward their opponents (1 = no trust at all, 9 = complete trust). (**B**) After completing the competition stage, participants played a modified version of a two-person public goods game (PGG). Participants were asked to indicate their own willingness to cooperate as well as their predictions about their opponents’ willingness to cooperate on a 9-point rating scale (1 = not at all to 9 = very willing). Meanwhile, participants had to decide how many tokens they wished to contribute to the public good and also predict how many tokens their opponents would contribute. MPCR, marginal per-capita return.

To answer these questions, we designed a novel experiment in which participants engaged in two stages: a competition stage and a cooperation stage (Fig. 1). In the first stage, once participants had competed against their opponents on a competitive task, they were given one of three types of outcome feedback (victory vs. defeat vs. uncertain competitive outcome), and compared with a group that performed the same task but framed in a manner without any competition (control). The choice of these sorts of outcome feedback was closely related to the design of a previous study^27^. Recent work reported the impact of competition on subsequent social behavior, in the absence of explicit relative performance feedback (uncertain competitive outcome)^31,32^. This is akin to a typical everyday competitive scenario in which people are very seldom explicitly informed about the outcome of their competitive performance. We thus included this type of outcome feedback to study its effect on the subsequent cooperation of participants toward their previous opponents. Performance-based competition has been found to regulate both self-evaluations of one’s own abilities such as self-confidence^33^ and interpersonal relationships such as closeness and trust^34,35^. Consequently, immediately after the outcome feedback, we asked participants to provide a rating of self-confidence as well as ratings of their feelings of interpersonal closeness and trust toward their opponents. In the second stage, alongside their previous opponent, participants performed a modified version of the two-person public goods game (PGG). We tested whether the level of cooperation would be affected by manipulating the marginal per-capita return for the PGG, thereby leading to three variable investment returns: participants faced a high risk for incurring personal costs, no risk for incurring personal costs, or a positive return from their contributions. Together with their actual cooperative behavior toward their opponents, participants also provided a number of self-reported measures, including an assessment of their willingness to cooperate preceding each decision and their predictions of both their opponents’ willingness to cooperate and the amount that their opponents would contribute.

The interdependence theory and the recent study described above^27^ suggest that cooperation in social dilemmas is driven by outcomes determined by the situation in combination with each individual’s social concerns and needs^36,37^. Therefore we predicted that after receiving negative (defeat) or uncertain competitive outcomes regarding their performance in competition, participants would subsequently decrease their willingness to cooperate with ex-opponents and decrease their own contribution to the public good. Moreover, previous work has revealed how outcome feedback affected subsequent decisions under risky situations. Individuals subsequently tend to take risks if risky choices offer a chance of receiving a potential gain^38–41^. Consequently, we predicted that the effect of defeat, and uncertain competitive outcome, would only be observed when participants' behavior would involve a high risk of incurring personal costs, but not when their behavior would involve no risk of incurring personal costs and promised a positive return from their contributions. Finally, according to the theory of planned behavior (TPB)^42^, human behavior is mainly guided by a favorable or unfavorable attitude toward the behavior. As a general rule, the more favorable the attitude, the more likely an individual should be to perform the behavior. This leads us to predict that participants’ willingness to cooperate may play a mediating role in how loss and uncertain competitive outcome affect their actual cooperative behavior toward their ex-opponents. Meanwhile, according to the interactive model of social value orientation, a partner’s trustworthiness promotes individuals’ cooperation^7^. Furthermore, there is a growing body of literature that emphasizes the role that trust toward counterparts plays in determining cooperation in conflicting situations^5,43,44^. Therefore we predicted that interpersonal relationships, as measured by trust but modulated by defeat or uncertain competitive outcomes, could act as a potential mediating variable.

## Results

### Pre-competition measures

We analyzed altruistic attitudes, cooperative and competitive orientation, and risk-taking scores using a one-way Analysis of Variance (ANOVA) with outcome feedback (victory vs. defeat vs. uncertain competitive outcome vs. control) as a between-participant factor. We did not find any significant differences in these personality traits among the four groups (altruistic attitudes: *F* (3,160) = 0.51, *p* = 0.674; competitive orientation: *F* (3,160) = 1.34, *p* = 0.263; cooperative orientation: *F* (3,160) = 0.40, *p* = 0.751; gamble risk-taking: *F* (3,160) = 0.10, *p* = 0.961; social-investment risk-taking: *F* (3,160) = 0.40, *p* = 0.753).

### Outcome feedback manipulation check and psychological reactions to outcome feedback

As a manipulation check, we analyzed ratings of perceived competition using a one-way ANOVA with outcome feedback (victory vs. defeat vs. uncertain competitive outcome vs. control) as a between-participant factor. We observed a significant effect of outcome feedback (*F* (3,160) = 18.71, *p* < 0.001, *η*2 p= 0.260). Bonferroni post hoc testing revealed that the defeated group (*M* = 6.05, *SE* = 0.28) reported significantly higher levels of perceived competition than the victory group (*M* = 4.12, *SE* = 0.28, *p* < 0.001, *d* = 1.06), suggesting that competition with outcome feedback manipulation was effective. Moreover, the uncertain competitive outcome group (*M* = 4.98, *SE* = 0.28) reported a significantly higher level of perceived competition than the control group (*M* = 3.17, *SE* = 0.28, *p* < 0.001, *d* = 0.99), indicating that competition without outcome feedback manipulation was effective. In addition, the defeated group reported significantly higher levels of perceived competition than the uncertain competitive outcome (*p* < 0.05, *d* = 0.59) and control groups (*p* < 0.001, *d* = 1.59). However, such ratings did not significantly differ between the uncertain competitive outcome and victory groups (*p* > 0.05, *d* = 0.47), and between the victory and control groups (*p* > 0.05, *d* = 0.52). Taken together, these results clearly demonstrate the effectiveness of competition with and without outcome feedback manipulation in our study.

**Figure 2:**
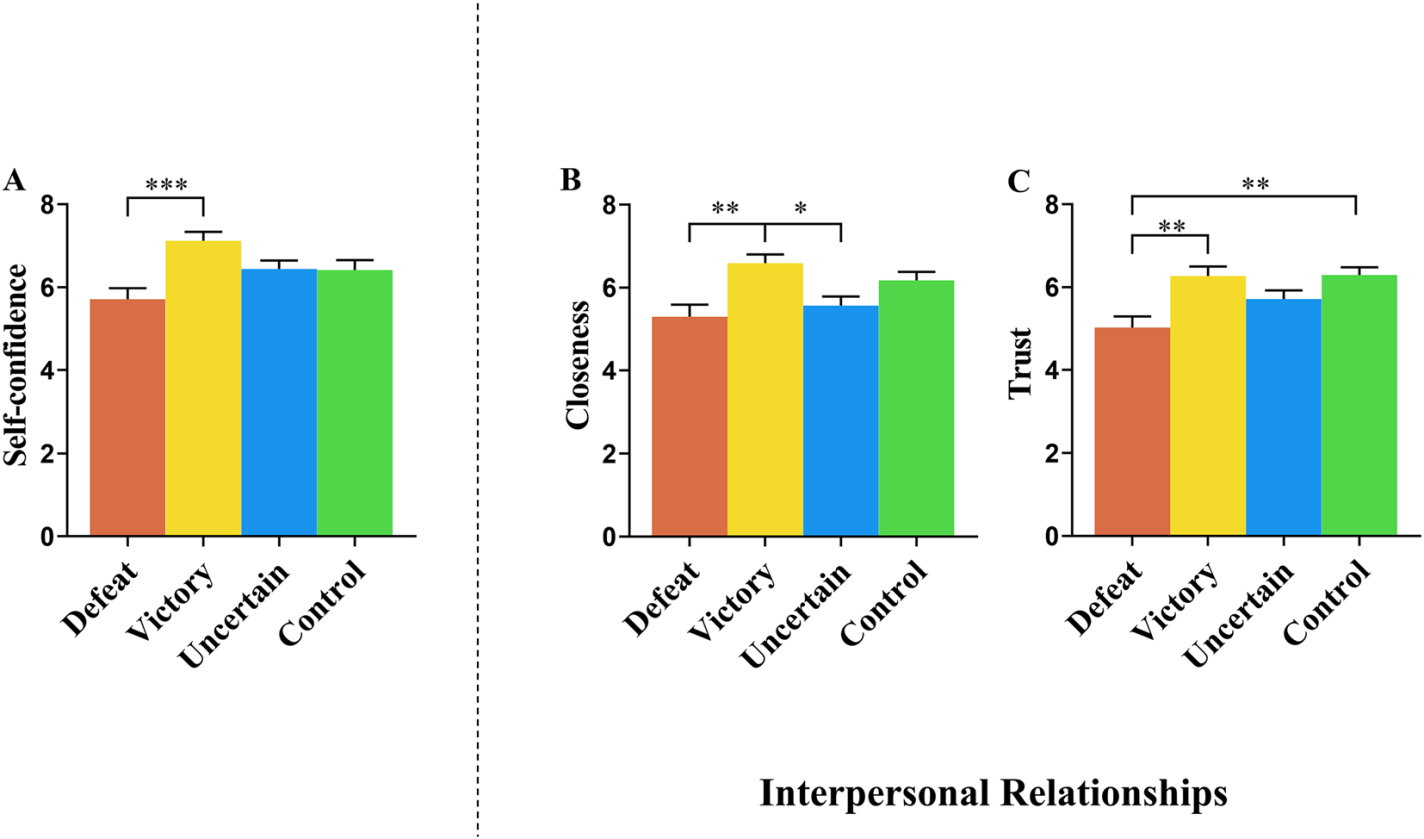
Effect of outcome feedback on participants’ self-reported measures. The graphs in (**A**–**C**) show mean ratings of self-confidence, closeness, and trust as a function of outcome feedback, respectively. *p < 0.05, **p < 0.01, ***p < 0.001. Error bars represent ± 1 SEM.

To examine psychological reactions to outcome feedback, we performed three separate one-way ANOVA on the ratings of self-confidence, closeness, and trust. Regarding self-confidence, we found a significant effect of outcome feedback (*F* (3, 160) = 6.17, *p* = 0.001, *η2 p* = 0.104) (Fig. 2A). Bonferroni post hoc testing revealed that the ratings were significantly lower in the defeated group (*M* = 5.71, *SE* = 0.23) than in the victory group (*M* = 7.12, *SE* = 0.23, *p* < 0.001, *d* = − 0.95), although there was no statistical difference in ratings between the defeated and the uncertain competitive outcome groups (*M* = 6.44, *SE* = 0.23, *p* > 0.05, *d* = -0.49) or between the defeated and control groups (*M* = 6.41, *SE* = 0.23, *p* > 0.05, *d* = − 0.47). This implies that self-confidence was decreased following negative compared to positive feedback. Meanwhile, such ratings did not significantly differ between the uncertain competitive outcome and control groups (*p* > 0.05, *d* = 0.02) as well as between the uncertain competitive outcome and victory groups (*p* > 0.05, *d* = − 0.46), indicating that self-confidence was not sensitive to competition in the absence of relative performance feedback. In addition, there was no significant difference between the victory and control groups (*p* > 0.05, *d* = 0.47). Concerning closeness, we found a significant effect of outcome feedback on ratings of perceived closeness (*F* (3, 160) = 6.27, *p* < 0.001, *η2 p* = 0.105) (Fig. 2B). Bonferroni post hoc testing revealed that the defeated group (*M* = 5.29, *SE* = 0.23) perceived less closeness to their opponents than the victory group (*M* = 6.59, *SE* = 0.23, *p* < 0.01, *d* = − 0.86) and the control group (*M* = 6.17, *SE* = 0.23, *p* = 0.05, *d* = − 0.59), although there was no significant difference in ratings between the defeated and the uncertain competitive outcome groups (*M* = 5.56, *SE* = 0.23, *p* > 0.05, *d* = − 0.18). Moreover, both the uncertain competitive outcome and control groups did not significantly differ in ratings of perceived closeness (*p* > 0.05, *d* = − 0.41), although there was a significant difference in such ratings between the uncertain competitive outcome and victory groups (*p* < 0.05, *d* = − 0.68). These results suggest that perceived closeness to opponents was susceptible to competition-outcome feedback rather than competition participation without relative performance feedback. In addition, we did not observe a significant difference in ratings between the victory and control groups (*p* > 0.05, *d* = 0.28). Finally, with regard to trust, a significant effect of outcome feedback on ratings of interpersonal trust was also found (*F* (3, 160) = 7.13, *p* < 0.001, *η2 p* = 0.118) (Fig. 2C). Bonferroni post hoc testing indicated that the defeated group (*M* = 5.02, *SE* = 0.22) rated their opponents less trustworthy than the victory group (*M* = 6.27, *SE* = 0.22, *p* < 0.01, *d* = − 0.87) and the control group (*M* = 6.29, *SE* = 0.22, *p* < 0.01, *d* = -0.89), while no such significant difference was found between the defeated and uncertain competitive outcome groups (*M* = 5.71, *SE* = 0.22 *p* > 0.05, *d* = − 0.48). There was not a significant difference in ratings of trust between the uncertain competitive outcome and control groups (*p* > 0.05, *d* = − 0.41), between the uncertain competitive outcome and victory groups (*p* > 0.05, *d* = − 0.39) or between the victory and control groups (*p* > 0.05, *d* = − 0.02).

These findings suggest that interpersonal trust was modulated by competition-outcome feedback rather than competition participation without relative performance feedback. They consistently demonstrate that self-evaluation, as captured by self-confidence, and interpersonal relationships, as measured by closeness and trust, were susceptible to competition with outcome feedback rather than competition without outcome feedback.

### The effects of competition outcome on risky cooperation

#### Willingness to cooperate

**Figure 3:**
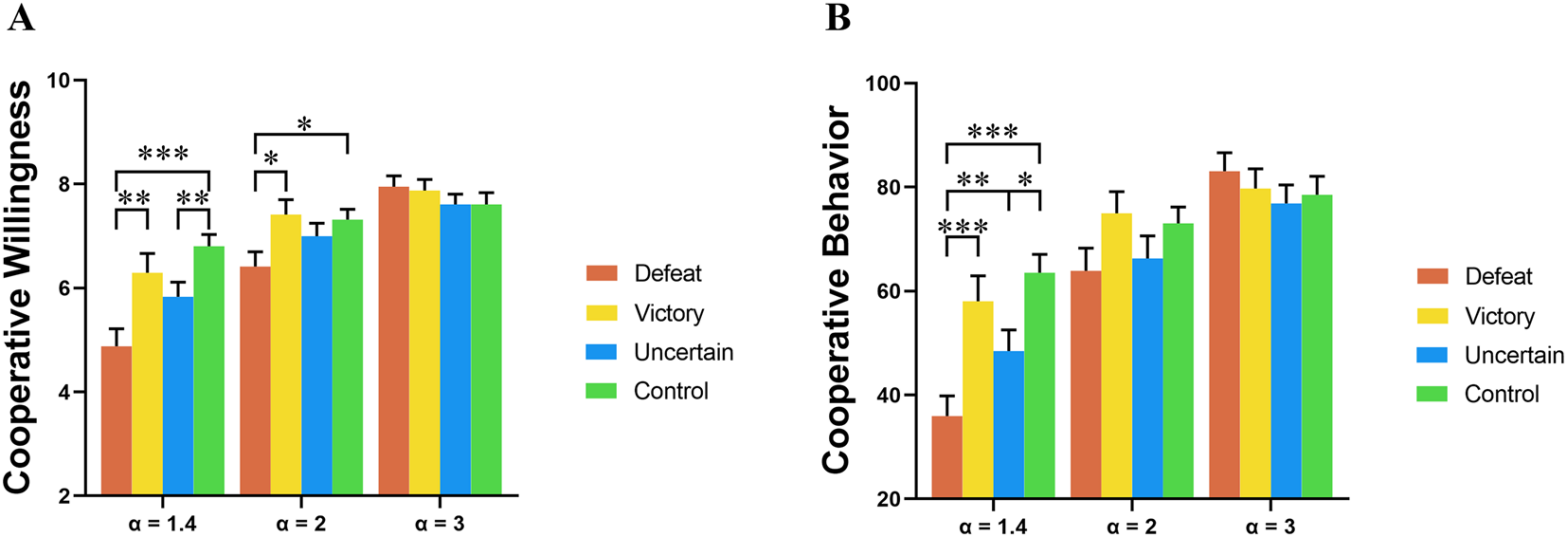
Effects of outcome feedback and the MPCR on participants’ willingness to cooperate and their cooperative behavior. With regard to participants’ willingness to cooperate with their opponents (**A**) and their cooperative behavior (**B**), an interaction between outcome feedback and the MPCR (α) occurred. *p < 0.05, **p < 0.01, ***p < 0.001. Error bars represent ± 1 SEM.

We analyzed ratings of willingness to cooperate using a three-way mixed ANOVA with outcome feedback (victory vs. defeat vs. uncertain competitive outcome vs. control) as a between-participant factor, person (self vs. opponent), and MPCR (α = 1.4 vs. α = 2 vs. α = 3) as the within-participant factor. We observed a significant main effect of both outcome feedback (*F* (3,160) = 3.81, *p* = 0.011, *η2 p* = 0.067) and MPCR (*F* (2, 320) = 89.97, *p* < 0.001, *η2 p* = 0.360). More importantly, these two main effects were further qualified by a significant interaction between them (*F* (6,320) = 6.04, *p* < 0.001, *η2 p* = 0.102) (Fig. 3A). A simple effects analysis using Bonferroni-adjusted comparisons revealed that, when there was a high risk of incurring personal costs (α = 1.4), the defeated group (*M* = 4.77, *SE* = 0.31) showed less willingness to cooperate with opponents than the victory group (*M* = 6.10, *SE* = 0.31, *p* < 0.01) and control group (*M* = 6.74, *SE* = 0.31, *p* < 0.001). This finding indicates that participants’ willingness to cooperate with opponents was significantly reduced after they received negative feedback compared to positive feedback in the competition. Likewise, in the same context, the uncertain competitive outcome group (*M* = 5.52, *SE* = 0.31) also showed less willingness to cooperate with opponents than the control group (*p* < 0.01). This suggests that participants’ willingness to cooperate with opponents was also decreased after experiencing competition in comparison with experiencing no competition. Tests for other comparisons did not show any significant difference (uncertain competitive outcome vs. victory, *p* > 0.05; victory vs. control, *p* > 0.05; defeat vs. the uncertain competitive outcome,* p* > 0.05). In contrast, when there was no risk of personal loss, but potential personal gain, depending on the opponent’s contribution (α = 2), the defeated group (*M* = 6.43, *SE* = 0.24) still exhibited less willingness to cooperate with opponents than the victory group (*M* = 7.28, *SE* = 0.24, *p* < 0.05) and control group (*M* = 7.16, *SE* = 0.24, *p* < 0.05), but no such significant difference in ratings occurred between the uncertain competitive outcome (*M* = 6.63, *SE* = 0.24) and control groups (*M* = 7.16, *SE* = 0.24, *p* > 0.05). These results suggest that participants’ willingness to cooperate with opponents was only sensitive to competition-outcome feedback but not competition without relative performance feedback, when there was no risk of personal loss but potential personal gain, depending on the opponent’s contribution (α = 2). Tests for other comparisons did not show any significant difference (uncertain competitive outcome vs. victory, *p* > 0.05; victory vs. control, *p* > 0.05; defeat vs. the uncertain competitive outcome,* p* > 0.05). Furthermore, when there was a positive return from participants’ contributions (α = 3), the defeated (*M* = 7.85, *SE* = 0.21) and victory groups (*M* = 7.76, *SE* = 0.21) showed similar levels of willingness to cooperate with opponents (*p* > 0.05). Furthermore, the uncertain competitive outcome (*M* = 7.35, *SE* = 0.21) and control groups (*M* = 7.53, *SE* = 0.21) did not significantly differ in ratings of willingness to cooperate (*p* > 0.05). These findings indicate that participants’ willingness to cooperate was not affected by competition-outcome feedback and competition without relative performance feedback when there is a positive return from their contributions (α = 3). No other significant differences in such ratings were found among other comparisons (defeat vs. uncertain competitive outcome, *p* > 0.05; defeat vs. control, *p* > 0.05; uncertain competitive outcome vs. victory, *p* > 0.05; victory vs control, *p* > 0.05). We observed a significant main effect of person (self vs. opponent) (*F* (1, 160) = 32.70, *p* < 0.001, *η2 p* = 0.170). Although no significant interaction between person and MCPR was found (*F* (2, 320) = 0.27, *p* > 0.05, *η2 p* = 0.002), there was a significant interaction between person and outcome feedback (*F* (3, 160) = 3.96, *p* = 0.009, *η2 p* = 0.069). Our simple effects analysis using Bonferroni-adjusted comparisons revealed that the victory and uncertain competitive outcome groups showed more willingness to cooperate with opponents when they rated their own willingness to cooperate (victory: *M* = 7.20, *SE* = 0.21; uncertain competitive outcome: *M* = 6.81, *SE* = 0.21), than when they predicted their opponents’ willingness to cooperate (victory: *M* = 6.89, *SE* = 0.21, *p* < 0.001; uncertain competitive outcome: *M* = 6.20, *SE* = 0.21, *p* < 0.01). However, the defeated and control groups rated their willingness to cooperate with opponents at similar levels when they rated their own willingness to cooperate (defeat: *M* = 6.42, *SE* = 0.21; control: *M* = 7.24, *SE* = 0.21) and when they predicted their opponents’ willingness to cooperate (defeat: *M* = 6.29, *SE* = 0.21, *p* > 0.05; control: *M* = 7.05, *SE* = 0.21, *p* > 0.05). Finally, there was no significant interaction among these three factors (*F* (6, 320) = 0.94, *p* > 0.05, *η2 p* = 0.017). See the supplementary material for further details of the results summarized above.

#### Cooperative behavior

We analyzed cooperative behavior using a three-way mixed ANOVA. Although there was no significant main effect of outcome feedback (*F* (3, 160) = 2.41, *p* > 0.05, *η2 p* = 0.043), there was a significant main effect of MPCR (*F* (2, 320) = 99.39, *p* < 0.001, *η2 p* = 0.383). Furthermore, there was a significant interaction between outcome feedback and MPCR (*F* (6, 320) = 6.40, *p* < 0.001, *η2 p* = 0.107). Our simple effects analysis using Bonferroni-adjusted comparisons revealed that only when there was a high risk of incurring personal costs (α = 1.4) would the defeated group (*M* = 34.15, *SE* = 3.83) contribute significantly less to the public good than the victory group (*M* = 55.85, *SE* = 3.83, *p* < 0.001) and control group (*M* = 61.28, *SE* = 3.83, *p* < 0.001), indicating that participants behaved less cooperatively toward opponents after they received negative feedback compared to positive feedback in the competition. The uncertain competitive outcome group (*M* = 49.52, *SE* = 3.83) contributed significantly less to the public good than the control group (*p* < 0.05) in the same context, suggesting that participants behaved less cooperatively toward opponents after experiencing competition in comparison with experiencing no competition. There was also a significant difference in contribution between the defeated and uncertain competitive outcome groups (*p* < 0.01). Tests for other comparisons did not show any significant difference (uncertain competitive outcome vs. victory, *p* > 0.05; victory vs. control, *p* > 0.05) (Fig. 3B). In contrast, when there was no risk of personal loss but potential personal gain, depending on the opponent’s contribution (α = 2), there was no significant difference between the defeated (*M* = 62.07, *SE* = 3.85) and victory groups (*M* = 71.52, *SE* = 3.85, *p* > 0.05) or between the uncertain competitive outcome (*M* = 64.96, *SE* = 3.85) and control groups (*M* = 69.94, *SE* = 3.85, *p* > 0.05). Tests for other comparisons did not show any significant difference (defeat vs. uncertain competitive outcome, *p* > 0.05; defeat vs. control, *p* > 0.05; uncertain competitive outcome vs. victory, *p* > 0.05; victory vs. control, *p* > 0.05). Similarly, when there was a positive return from participants’ contributions (α = 3), there was no significant difference between the defeated (*M* = 82.07, *SE* = 3.59) and victory groups (*M* = 77.93, *SE* = 3.59, *p* > 0.05) or between the uncertain competitive outcome (*M* = 75.38, *SE* = 3.59) and control groups (*M* = 76.65, *SE* = 3.59, *p* > 0.05). Tests for other comparisons did not reveal any significant difference (defeat vs. uncertain competitive outcome, *p* > 0.05; defeat vs. control, *p* > 0.05; uncertain competitive outcome vs. victory,* p* > 0.05; victory vs. control, *p* > 0.05). These results demonstrate that participants’ cooperative behavior toward opponents was not sensitive to competition-outcome feedback and competition without relative performance feedback both when there was no risk of personal loss but there was a potential personal gain, depending on the opponent’s contribution (α = 2) and when there is a positive return from participants’ contributions (α = 3). In addition, we observed a significant main effect of person (self vs. opponent) (*F* (1, 160) = 22.98, *p* < 0.001, *η2 p* = 0.126), showing that participants contributed significantly more to the public good when they had rated their own levels of cooperation (*M* = 66.88, *SE* = 1.59) compared to when they predicted their opponents’ levels of cooperation (*M* = 63.34, *SE* = 1.47). However, there was no significant interaction between person and outcome feedback (*F* (3, 160) = 1.43, *p* > 0.05, *η2 p* = 0.026) or MCPR (*F* (2, 320) = 1.52, *p* > 0.05, *η2 p* = 0.009). Finally, the interaction between outcome feedback, person, and MCPR was not significant (*F* (6, 320) = 0.72, *p* > 0.05, *η2 p* = 0.013). See the supplementary material for further details of the results summarized above.

### Potential mechanisms underlying effects of competition-outcome feedback and competition on cooperative behavior

Up to this point, we have observed that participants showed reduced trust toward opponents and decreased willingness to cooperate with opponents following negative feedback compared to positive feedback in the competition when there was a high risk of incurring personal costs (α = 1.4). In contrast, participants only showed a decreased willingness to cooperate with opponents following competition participation without relative performance feedback in the same context. In addition to theoretical considerations viewing trust and willingness to cooperate as potentially mediating variables^42^, these findings led us to speculate that both trust and willingness to cooperate may play a mediating role in how competition-outcome feedback affects participants’ cooperative behavior toward their ex-opponents. However, only willingness to cooperate may play a mediating role in how competition participation without relative performance feedback impacts participants’ cooperative behavior toward their ex-opponents. To address this issue, we conducted three separate mediation analyses in which either trust or willingness to cooperate were tested as mediating variables on the effect of competition-outcome feedback and in which only willingness to cooperate was tested as a mediating variable on the effect of competition participation. We obtained bias-corrected bootstrap confidence intervals for the indirect effects. In each model, a total of 10,000 bootstraps resamples were used to estimate confidence intervals.

**Figure 4:**
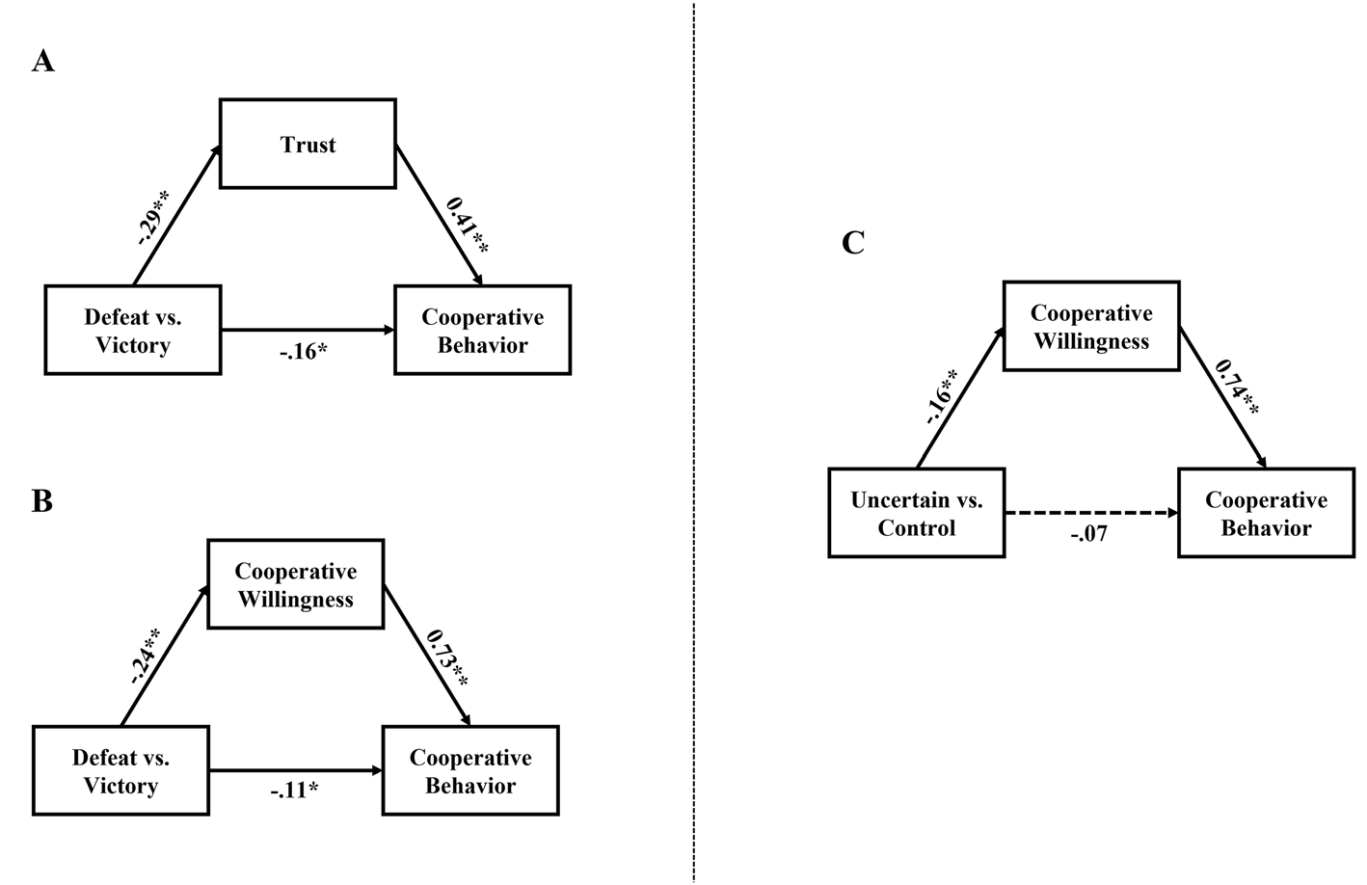
Mediation results. The effect of competition-outcome feedback on participants’ cooperative behavior toward their opponents is mediated through trust (**A**) and the willingness to cooperate (**B**). In contrast, the effect of competition without relative performance feedback on participants’ cooperative behavior toward their opponents is mediated through the willingness to cooperate (**C**). The model refers to behavior when there was a high risk of incurring personal costs (α = 1.4). Nonsignificant mediating variables were excluded for presentation clarity. Standardized regression coefficients (*B*) were presented on the significant paths as denoted by solid lines. No significant paths were represented by dash lines. *p < 0.05, **p < 0.01.

When comparing the defeated group with the victory group, we observed the presence of mediation by the level of trust participants felt toward opponents (Fig. 4A). This mediator showed a significant negative indirect effect (*B* = − 0.12, *SE* = 0.04, 95%CI = [− 0.20, − 0.06]). Specifically, defeat in the competition decreased trust in the opponents of participants (*B* = − 0.29, *SE* = 0.08, *p* < 0.001). This in turn led to lower levels of risky cooperative behavior in participants toward their opponents (*B* = 0.41, *SE* = 0.07, *p* < 0.001). Meanwhile, we also found the presence of mediation by participants’ willingness to cooperate with opponents (Fig. 4B). This mediator showed a significant negative indirect effect (*B* = − 0.17, *SE* = 0.06, 95%CI = [− 0.29, − 0.05]). Specifically, defeat in the competition decreased participants’ willingness to cooperate with their previous opponents (*B* = − 0.24, *SE* = 0.09, *p* < 0.01). This in turn led to lower levels of risky cooperative behavior toward their opponents (*B* = 0.73, *SE* = 0.05, *p* < 0.001).

When comparing the uncertain competitive outcome group with the control group, we observed cooperative behavior toward opponents to be mediated by participants’ willingness to cooperate (Fig. 4C). This mediator showed a significant negative indirect effect (*B* = − 0.12, *SE* = 0.05, 95%CI = [− 0.22, − 0.03]). Specifically, the uncertain competitive outcome was associated with a decrease in participants’ willingness to cooperate with their previous opponents (*B* = − 0.16, *SE* = 0.06, *p* < 0.01). This in turn decreased their cooperative behavior toward their opponents (*B* = 0.74, *SE* = 0.05, *p* < 0.001).

## Discussion

In this study, we show how prior competition-driven outcomes shape participants’ subsequent cooperative behavior toward their previous opponents in contexts involving different levels of risk for personal losses and gains. By examining the psychological reactions of participants to outcome feedback during competition, we demonstrated a detrimental impact of the defeat outcome compared to the victory outcome. Specifically, defeat compared to victory exerted a deleterious effect on the self-confidence of participants and on their subsequent interpersonal relationships with opponents as assessed by interpersonal closeness and trust. It has been proposed that the nature of one’s competitive interactions with others largely depends on one’s evaluation of competition-outcome feedback^45–50^. For example, prevailing over one’s opponent (victory) signals a relative competitive advantage (dominance), while conceding defeat signals a relative competitive disadvantage (deference)^51–53^. Thus, our results indicate that those who suffered from such a relative competitive disadvantage show reduced levels of subsequent cooperation with their opponents. By manipulating contexts in such a way as to produce a high risk of incurring personal costs, we also demonstrated that, relative to other groups, the defeated group was less inclined to cooperate with their opponents and contributed less to the public good. Our results add to a growing body of literature supporting the notion that individuals that suffer prior losses, be they financial^39,54^ or competitive^55^, become averse to making subsequent risky decisions that offer less chance of receiving a potential gain.

Furthermore, we found that relative to the control group, the uncertain competitive outcome group was less inclined to cooperate and contribute to the public good. This was despite the fact that this condition exerted no discernible effect on either their self-confidence or their relationships with opponents. This implies that there may be distinct psychological processes at play. This argument is supported by our mediation results. Thus, defeats had a negative effect on the subsequent cooperative behavior of participants via the mediating role of their own willingness to cooperate and the perceived trustworthiness of their opponents. In contrast, a comparable impact of competition participation with individual performance feedback (uncertain competitive outcome) on participants’ cooperative behavior was achieved only through the mediating role of participants’ willingness to cooperate. These findings not only provide further evidence supporting the theory of planned behavior (TPB)^42^, but also emphasize the role that trust plays toward counterparts in determining cooperation in conflicting situations^43,44^. Beyond this, these results demonstrate that participants’ willingness to cooperate, as a mediating factor, was not dependent upon the outcome regarding their relative performance. In contrast, the mediating role played by the trust of participants in their opponents relies on the explicit outcome regarding their relative performance. In this sense, relative performance feedback during competition seems to act as a signal for evaluating the trustworthiness of others in situations involving conflict such as competition. These findings are consistent with the claim that trust refers to expectations about the benevolence of another individual during cooperation^7,44,56,57^.

In addition to the findings outlined above, our study provides new insights into how behavioral changes induced by initial competitive experiences, determined by defeats and uncertain competitive outcomes, interact with the motive of participants’ self-interest to form their decisions to cooperate with their opponents. Specifically, when there was no risk of personal loss, but only gains, depending on the contributions made by their opponents, only the defeated group remained less inclined to cooperate with their opponents (compared to the victory and control groups). However, we did not observe such an effect on participants’ actual contributions to the public good in the same context. Those who suffered defeats during the competitive phase still preferred to prioritize their own interests by offering cooperation to take advantage of the opportunity for personal gain. Even though they showed less willingness to cooperate, these participants seemingly felt obligated to take the risk of their previous opponents free-riding, and themselves receiving nothing in return, and thus only benefitting their counterparts. Moreover, when the context implied that contributions by participants would benefit both themselves and their opponents, we observed that outcome feedback had no significant impact on either participants’ willingness to cooperate or their cooperative behavior toward their previous opponents. This indicates that the subsequent behavior of participants toward their opponents tends to be self-serving or mutually beneficial. These findings extend results from recent studies that stress the need to take competition outcomes into account when attempting to understand how human competitors behave in the context of later risky cooperation^27,58,59^, an area that has been extensively investigated in non-human animals^60,61^.

### Limitations

In spite of our promising findings, there are several potential limitations of this study that need to be pointed out. First, empirical research on the relation between wealth and consumption has demonstrated that increased aggregate wealth results in an increase in aggregate consumption^62^. For this reason, in the context of risky cooperation, participants’ behavior toward their opponents may be confounded by such a wealth effect arising from the competition stage. Thus, participants in the defeated group only received a participation fee of ￥10, while winners received a participation fee of ￥10 and a bonus of ￥10. Future research should take this aspect into account. Second, our current study only used a two-player PGG, which provides the simplest environment to assess how participants behave toward previous opponents within the context of risky cooperation. For this reason, the “generalizability” of these findings to multi-player games that simulate more real-life interactions remains unclear. Future research should extend our examination by taking the step from two-player games to multi-player games. Third, no ‘pure’ control of not knowing the partner’s identity was employed in our study, which may temper our findings. Future research with this additional control condition should be considered. Fourth, the present study only employed interpersonal competition. However, it has been shown that group-based competition, which is a distinct form of competition, plays an important role in shaping individuals’ social behavior^55,63,64^. As a result, it would be interesting to explore the similarities and differences in the role of these two forms of competition on individuals’ subsequent risky cooperation. Fifth, there has been a hot-button debate regarding the underlying causal inference problems with conventional mediation analysis^65^. Although randomized experiments have been claimed to be one way to rule out both confounding and reverse causality that often tempers claims about the causal role of a mediating variable, future research following the suggested practice would strengthen the clarity of the causal claims based on the mediation results. Sixth, a recent study reported that younger adults made superior strategic allocations and won more frequently in competition against older adults than in competition against opponents of similar age^66^. This would deepen our understanding of how exposure to competitive interactions shapes subsequent risky cooperation with ex-opponents if future research can take age-related differences in strategic behavior toward opponents into account. Last but not least, the sample size in this study was calculated by the power analysis. Although such power is warranted to validate the conclusions of the study, we still believe that future research with a larger sample size may still provide additional insights into this topic.

## Conclusion

To conclude, we provide novel evidence showing that individuals’ cooperative decisions toward their opponents vary depending on outcome-based feedback of their prior competition. Negative feedback (defeat) compared to positive feedback (victory) had a negative effect on the subsequent cooperative behavior toward opponents via the mediating role of willingness to cooperate and the perceived trustworthiness of opponents. In contrast, the comparable impact of competition participation with individual performance feedback on participants’ cooperative behavior toward opponents was achieved only through the mediating role of participants’ willingness to cooperate. Rather than depending on the person through whom the decisions were made, this effect operates in a context-dependent manner. Our results add to emerging research showing that the nature of cooperative interactions between individuals can vary depending on their evaluation of the outcome of prior competitive interactions.

## Materials and methods

### Participants

This experiment was performed in the Experimental Laboratory at the Department of Psychology, University of Nanjing. To ensure adequate power in the current research, we performed a power analysis using R to determine the sample size of our study with a mixed three-way ANOVA design. We calculated that to maintain 80% power for an alpha level of 0.05, then the sample size needed to detect a medium effect size (Cohen’s *f* = 0.28)^67,68^ was at least 31 participants per condition. We based the assumption of the medium effect size on previous research regarding the impact of competition-outcome feedback on risky cooperation^27^. In fact, we recruited 41 participants per condition in order to ensure the robustness of the present study. In total, 164 adults (87 females, *M*_age_ = 20.82 years, *SD* = 2.13), recruited from the University of Nanjing psychology participant pool, took part in the study. All participants gave written informed consent prior to participation. At the end of the study, participants received the payment. The final payment included the participation fee, the bonus fee from the competition stage depending on whether they won the competition, and contributions from the cooperation stage. This study was approved by the Institutional Review Board of Nanjing University. This study has been performed in accordance with the ethical standards laid down in the 1964 Declaration of Helsinki and its later amendments. Following the “21-word solution”^69^, we declare that “we report how we determined our sample size, all data exclusions (if any), all manipulations, and all measures in the study”.

### Procedure

#### Pre-competition measures

We administered several questionnaires with the aim of controlling for potential individual differences and how they might relate to task performance^70^. These questionnaires comprised the Chinese version of the Self-Report Altruism Scale (C-SRA) to examine altruistic attitudes among participants (Cronbach’s α = 0.80)^71^, the Chinese version of the Cooperative and Competitive Orientation Scale (C-CCO) to assess how inclined participants were to compete (Cronbach’s α = 0.71)^72^, and the Chinese version of the Domain-Specific Risk-Taking Scale (C-DOSPERT) to test risk-taking inclinations among participants (Cronbach’s α = 0.75)^73^.

#### Competition with outcome feedback manipulation

We used a between-participants design to manipulate outcome feedback. Participants were randomly assigned to receive one of four types of outcome feedback (victory vs. defeat vs. uncertain competitive outcome vs. no competition (control)). We used a standard version of the Deese–Roediger–McDermott (DRM) paradigm^74,75^ as the competitive task, which is the same as that described in our recently published article^76^. More specifically, this task includes three phases: a study phase, a distractor phase, and a test phase. During the study phase, participants were asked to learn ten lists of 15 related words with each list strongly associated with an absent critical lure. Prior to being tested, participants performed the distractor phase. This was comprised of 5-min of buffer activities in which they were asked to solve simple arithmetic problems (e.g., 3 × 4 + 5 = ?). Immediately after this, participants performed the test phase. This comprised a recognition memory test in which participants were required to respond “yes” or “no” to each word from a 60-item test list, depending on whether they believed the word appeared in a list from the study phase. The list contained 30 target words from the study phase as well as 10 related distractor words and 20 unrelated distractor words (Fig. 1A). Participants performed one round of the DRM task against their opponents.

To stimulate competition, participants were playing against an opponent on the DRM paradigm via two connected computers. To intensify their sense of competition, we also informed them that if they outperformed their opponent, they would get a bonus payment of ￥10. Otherwise, they would only obtain the standard payment for their participation in the study (￥10). At the end of the competition, participants were immediately given information about their performance. Specifically, they received one of three types of outcome feedback (victory vs. defeat vs. uncertain competitive outcome). Regarding the uncertain competitive outcome, participants were informed of their own task performance but had no information about the performance of their opponents. In this way, participants had no way to directly evaluate their performance relative to their opponents. For the other two outcome feedbacks, participants received them at the end of the competition that was appropriate to their group, i.e., that they had won (“you win”) or lost (“you lose”) against their opponents. Due to technical challenges, the outcome feedback that participants received was not based on comparing participants’ performance with their opponents’ performance but pre-determined. Such outcome feedback manipulation inevitably raises a concern with respect to deception of participants^77^. However, we believe that it does not undermine the quality of our results. Outcome feedback was counterbalanced between participants to prevent order effects. After receiving outcome feedback, participants were asked to provide a rating on a nine-point Likert scale of their perceived competition (the intensity they felt about the competition) (1 = not at all, 9 = extreme), self-confidence (1 = not at all confident, 9 = completely confident), the degree of closeness that they felt toward their opponents (1 = not at all close, 9 = completely close) and the level of trust that they felt toward their opponents (1 = not at all, 9 = complete trust) (Fig. 1A).

In the control “no competition” condition, participants were told that they and another person would perform the DRM paradigm independently. As such, no competition occurred between them and they were asked to perform the task as well as possible. Once participants had completed the task, they were given feedback about their own task performance. They were informed that, if their performance level ranked above a predetermined criterion, then they would receive a bonus payment of ￥10. Otherwise, they would only get the standard payment for participating in the experiment. However, like the three competition conditions, immediately after performance feedback, participants rated on a 9-point scale the extent to which they perceived competition, self-confidence, their closeness to the other individuals as well as the level of trust they felt toward them.

#### Risky cooperation: two-person Public Goods Game

After completing the competition stage, participants played a one-shot, two-player public goods game (PGG). This provides the simplest environment to assess how participants behaved toward their opponents within the context of risky cooperation. At the start of the PGG, participants were informed of their endowment (100 tokens with an exchange value of 10 tokens per￥1), that of the player with whom they were matched, and whether or not this person had been allocated their endowment. First, participants were asked to indicate their own willingness to cooperate as well as their predictions about their opponents’ willingness to cooperate on a 9-point rating scale (1 = not at all to 9 = very much respectively). Given that there is some evidence showing differences in self-other decision making^78,79^, we additionally measured participants’ predictions about their opponents’ willingness to cooperate and cooperative behavior, to explore the possible influence of self-others discrepancies in participants’ decisions to cooperate. We counterbalanced the order of such ratings across participants to prevent order effects.

Participants had to decide how many tokens they wished to contribute to the public good and predict how many their opponents would contribute. The order of these predictions was also counterbalanced across participants to prevent order effects. The number of tokens that had been donated to the public good was then multiplied by a factor that was known to participants before they contributed. Multiplying contributions by a factor larger than the number of players can guarantee a positive return from participants’ contributions, which means that allocations to the public good can be purely based on their self-interest. This produced a final amount that was then evenly divided between the players irrespective of who had contributed the most. As such, personal earnings comprised the sum of tokens that participants did not contribute to the public good plus their share of the payment from the public good. We applied three α: 1.4, 2, and 3 to the amounts contributed. In this sense, our modified PGG allowed participants to make decisions about cooperation with their opponents when their cooperative behavior involved either a high risk of incurring personal costs, no risk of incurring personal costs, or mutual gains. Specifically, when α = 1.4, participants run a high risk of personal costs. For example, a contribution of 40 tokens, when multiplied by 1.4, would result in an overall amount of 56. Thus, if the opponent decides to contribute nothing, the participant will receive less than their initial contribution (56/2 = 28). When α = 2, there is no risk but gains are not guaranteed. For example, a contribution of 40 tokens, when multiplied by 2, would only bring about an overall amount of 80. Thus, if the opponent decides to contribute nothing, the participant will receive the same as their initial contribution (80/2 = 40). In this situation, the opponent would benefit from the participant’s contribution. When α = 3 however, risk is again 0 but gain this time is guaranteed, regardless of whether the opponent decides to contribute. For example, a contribution of 40 tokens, when multiplied by 3, would result in an overall amount of 120. Thus, even though the opponent decides to contribute nothing, both the participant and the opponent will still receive more than the participant’s initial contribution (120/2 = 60) (Fig. 1B).

## Supplementary Information

Supplementary Information.

## Data Availability

The raw datasets containing a codebook are available at https://osf.io/qy9e7/.
