# Supplementary material for: Spillover effects of competition outcome on future risky cooperation
Source: Sci Rep. 2023 Apr 4;13:5535. doi: 10.1038/s41598-023-32523-6 (PMC10073108; doi:10.1038/s41598-023-32523-6)
Supplement: Supplementary file 1 — Supplementary Information. [file 41598_2023_32523_MOESM1_ESM.docx]

**Supplementary materials**

**Spillover effects of competition outcome on future risky cooperation**

Yansong Li^1,2,3^***^*^***, Zhenliang Liu^6^, Yuqian Wang^2^, Edmund Derrington^5^, Frédéric Moisan^4^, Jean-Claude Dreher^5^

^1^ Department of Radiology, The Affiliated Drum Tower Hospital of Nanjing University Medical School, Nanjing, China

^2^ Reward, Competition, and Social Neuroscience Lab, Department of Psychology, School of Social and Behavioral Sciences, Nanjing University, Nanjing, China

^3^ Institute for Brain Sciences, Nanjing University, Nanjing, China

^4^ EM-Lyon and GATE CNRS, Ecully, France

^5^ Institute of Cognitive Science Marc Jeannerod, CNRS, Lyon, France

^6^ Nanjing University, Nanjing, China

**Appendix: Results**

**Willingness to cooperate**

We analyzed ratings of willingness to cooperate using a three-way mixed ANOVA with outcome feedback (victory vs. defeat vs. uncertain competitive outcome vs. control) as a between-participant factor, person (self vs. opponent), and MPCR (1.4 vs. 2 vs. 3) as the within-participant factor. We observed a significant main effect of outcome feedback (*F* (3,160) = 3.81, *p* = .011, *η2 p* = .067). Bonferroni post hoc testing revealed that the defeated group (*M* = 6.35, *SE* = .20) showed less willingness to cooperate with opponents than both the victory (*M* = 7.05, *SE* = .20, *p* < .05, *d* = -.22) and control groups (*M* = 7.15, *SE* = .20, *p* < .01, *d* = -.25), although both the defeated and uncertain competitive outcome groups (*M* = 6.50, *SE* = .20) did not significantly differ in such ratings (*p* > .05, *d* = -.05). Moreover, the uncertain competitive outcome group showed less willingness to cooperate with opponents than the control group (*p* < .05, *d* = -.21), while the uncertain competitive outcome and victory groups did not significantly differ in such ratings (*p* > .05, *d* = -.17). Also, the victory and control groups did not differ each other (*p* > .05, *d* = -.03). We also found a significant main effect of MPCR (*F* (2, 320) = 89.97, *p* < .001, *η2 p*= .360). Bonferroni post hoc testing revealed that participants exhibited less willingness to cooperate when there was a high risk for incurring personal costs (α = 1.4) (*M* = 5.78, *SE* = .15) than when there was no risk of personal loss but potential personal gain, depending on the opponent’s contribution (α = 2) (*M* = 6.88, *SE* = .12, *p* < .001, *d* = -.35) and when there is a positive return from participants’ contributions from participants’ contributions (α = 3) (*M* = 7.63, *SE* = .11, *p* < .001, *d* = -.59). Participants also showed less willingness to cooperate when there was no risk of personal loss but potential personal gain, depending on the opponent’s contribution (α = 2) than when there is a positive return from participants’ contributions (α = 3) (*p* < .001, *d* = -.24). More importantly, these two main effects was further qualified by a significant interaction between them (*F* (6,320) = 6.04, *p* < .001, *η2 p*= .102) (**Fig** **3A**). A simple effects analysis using Bonferroni-adjusted comparisons revealed that, when there was a high risk of incurring personal costs (α = 1.4), the defeated group (*M* = 4.77, *SE* = .31) showed less willingness to cooperate with opponents than the victory (*M* = 6.10, *SE* = .31, *p* < .01) and control groups (*M* = 6.74, *SE* = .31, *p* < .001). This finding indicates that participants’ willingness to cooperate with opponents was significantly reduced after they received negative feedback compared to positive feedback in the competition. Likewise, under the same context, the uncertain competitive outcome group (*M* = 5.52, *SE* = .31) also showed less willingness to cooperate with opponents than the control group (*p* < .01). This suggests that participants’ willingness to cooperate with opponents was also decreased after experiencing competition in comparison with experiencing non-competition. Test for other comparisons did not show any significant difference (uncertain competitive outcome vs. victory, *p* > .05; victory vs. control, *p* > .05; defeat vs. the uncertain competitive outcome, *p* > .05). In contrast, when there was no risk of personal loss but potential personal gain, depending on the opponent’s contribution (α = 2), the defeated group (*M* = 6.43, *SE* = .24) still exhibited less willingness to cooperate with opponents than the victory (*M* = 7.28, *SE* = .24, *p* < .05) and control groups (*M* = 7.16, *SE* = .24, *p* < .05), while no such significant difference in ratings between the uncertain competitive outcome (*M* = 6.63, *SE* = .24) and control groups (*M* = 7.16, *SE* = .24, *p* > .05). These results suggest that participants’ willingness to cooperate with opponents was only sensitive to competition-outcome feedback rather than the competition without relative performance feedback when there was no risk of personal loss but potential personal gain, depending on the opponent’s contribution (α = 2). Test for other comparisons did not show any significant difference (uncertain competitive outcome vs. victory, *p* > .05; victory vs. control, *p* > .05; defeat vs. the uncertain competitive outcome, *p* > .05). Furthermore, when there is a positive return from participants’ contributions (α = 3), the defeated (*M* = 7.85, *SE* = .21) and victory groups (*M* = 7.76, *SE* = .21) showed their willingness to cooperate with opponents at similar levels (*p* > .05). Furthermore, the uncertain competitive outcome (*M* = 7.35, *SE* = .21) and control groups (*M* = 7.53, *SE* = .21) did not significantly differ in ratings of willingness to cooperate (*p* > .05). These findings indicated that participants’ willingness to cooperate was not affected by competition-outcome feedback and competition without relative performance feedback when there is a positive return from their contributions (α = 3). No other significant differences in such ratings were found among other comparisons (defeat vs. uncertain competitive outcome, *p* > .05; defeat vs. control, *p* > .05; uncertain competitive outcome vs. victory, *p* > .05; victory vs control, *p* > .05). Meanwhile, we observed a significant main effect of person (self vs. opponent) (*F* (1, 160) = 32.70, *p* < .001, *η2 p* = .170). Although no significant interaction between person and MCPR was found (*F* (2, 320) = .27, *p*> .05, *η2 p*= .002), there was a significant interaction between person and outcome feedback (*F* (3, 160) = 3.96, *p* = .009, *η2 p*= .069). Our simple effects analysis using Bonferroni-adjusted comparisons revealed that the victory and uncertain competitive outcome groups showed more willingness to cooperate with opponents when they rated their own willingness to cooperate (victory: *M* = 7.20, *SE* = .21; uncertain competitive outcome: *M* = 6.81, *SE* = .21) than when they predicted their opponents’ willingness to cooperate (victory: *M* = 6.89, *SE* = .21, *p* < .001; uncertain competitive outcome: *M* = 6.20, *SE* = .21, *p* < .01). However, the defeated and control groups showed their willingness to cooperate with opponents at similar levels when they rated their own willingness to cooperate (defeat: *M* = 6.42, *SE* = .21; control: *M* = 7.24, *SE* = .21) than when they predicted their opponents’ willingness to cooperate (defeat: *M* = 6.29, *SE* = .21, *p* > .05; control: *M* = 7.05, *SE* = .21, *p* > .05). Finally, there was not a significant interaction among these three factors (*F* (6, 320) = .94, *p* > .05, *η2 p*= .017).

**Cooperative behavior**

We also analyzed cooperative behavior using a three-way mixed ANOVA. Although no significant main effect of outcome feedback (*F* (3, 160) = 2.41, *p* > .05, *η2 p* = .043), there was a significant main effect of MPCR (*F* (2, 320) = 99.39, *p* < .001, *η2 p*= .383). Bonferroni post hoc testing revealed that participants contribute significantly less to the public good when there was a high risk of incurring personal costs (α = 1.4) (*M* = 50.20, *SE* = 1.91) than when there was no risk of personal loss but potential personal gain, depending on the opponent’s contribution (α = 2) (*M* = 67.13, *SE* = 1.93, *p* < .001, *d* = -.66) and when there is a positive return from their contributions (α = 3) (*M* = 78.01, *SE* = 1.79, *p* < .001, *d* = -1.09). Participants also showed less willingness to cooperate when there was no risk of personal loss but potential personal gain, depending on the opponent’s contribution (α = 2) than when there is a positive return from their contributions (α = 3) (*p* < .001, *d* = -.43). Furthermore, there was a significant interaction between outcome feedback and MPCR (*F* (6, 320) = 6.40, *p* < .001, *η2 p*= .107). Our simple effects analysis using Bonferroni-adjusted comparisons revealed that only when there was a high risk of incurring personal costs (α = 1.4) would the defeated group (*M* = 34.15, *SE* = 3.83) contribute significantly less to the public good than the victory (*M* = 55.85, *SE* = 3.83, *p* < .001) and control groups (*M* = 61.28, *SE* = 3.83, *p* < .001), indicating that participants behaved less cooperatively toward opponents after they received negative feedback compared to positive feedback in the competition. Meanwhile, the uncertain competitive outcome group (*M* = 49.52, *SE* = 3.83) contributed significantly less to the public good than the control group (*p* < .05) in the same context, suggesting that participants behaved less cooperatively toward opponents after experiencing competition in comparison with experiencing non-competition. There was also a significant difference in contribution between the defeated and uncertain competitive outcome groups (*p* < .01). Test for other comparisons did not show any significant difference (uncertain competitive outcome vs. victory, *p* > .05; victory vs. control, *p* > .05) (**Fig** **3B**). In contrast, when there was no risk of personal loss but potential personal gain, depending on the opponent’s contribution (α = 2), there was not a significant difference between the defeated (*M* = 62.07, *SE* = 3.85) and victory groups (*M* = 71.52, *SE* = 3.85, *p* > .05) as well as between the uncertain competitive outcome (*M* = 64.96, *SE* = 3.85) and control groups (*M* = 69.94, *SE* = 3.85, *p* > .05). Test for other comparisons did not show any significant difference (defeat vs. uncertain competitive outcome, *p* > .05; defeat vs. control, *p* > .05; uncertain competitive outcome vs. victory, *p* > .05; victory vs. control, *p* > .05). Similarly, when there is a positive return from participants’ contributions (α = 3), there was not a significant difference between the defeated (*M* = 82.07, *SE* = 3.59) and victory groups (*M* = 77.93, *SE* = 3.59, *p* > .05) as well as between the uncertain competitive outcome (*M* = 75.38, *SE* = 3.59) and control groups (*M* = 76.65, *SE* = 3.59, *p* > .05). Test for other comparisons did not reveal any significant difference (defeat vs. uncertain competitive outcome, *p* > .05; defeat vs. control, *p* > .05; uncertain competitive outcome vs. victory, *p* > .05; victory vs. control, *p* > .05). These results demonstrated that participants’ cooperative behavior toward opponents was not sensitive to competition-outcome feedback and competition without relative performance feedback both when there was no risk of personal loss but potential personal gain, depending on the opponent’s contribution (α = 2) and when there is a positive return from participants’ contributions (α = 3). In addition, we observed a significant main effect of person (self vs. opponent) (*F* (1, 160) = 22.98, *p* < .001, *η2 p*= .126), showing that participants contributed significantly more to the public good when they had rated their own levels of cooperation (*M* = 66.88, *SE* = 1.59) compared to when they predicted their opponents’ levels of cooperation (*M* = 63.34, *SE* = 1.47). However, there is not a significant interaction between person and outcome feedback (*F* (3, 160) = 1.43, *p* > .05, *η2 p*= .026) as well as MCPR (*F* (2, 320) = 1.52, *p* > .05, *η2 p*= .009). Finally, the interaction among outcome feedback, person, and MCPR was not significant (*F* (6, 320) = 0.72, *p* > .05, *η2 p*= .013). See the supplementary material for further details of the results summarized above.

**Appendix: Instructions**

**Instruction for competition condition**

Hello, welcome to the experiment.

Let me briefly introduce the basic situation of the experiment. The current experiment consists of two stages.

**The first stage:**

The first stage is a competition experiment in which you need to perform a memory task. We will match you with another player for an online competition. Your goal is to outperform the opponent. You need to peform the task as good as possible. The memory task is divided into three phases: learning, distractor and recognition phase. In the learning stage, you need to learn a series of word phrases and try to remember as many as possible. After learning, you need to perform some simple arithmetic operations. Then, in the recognition stage, you need to judge whether some words were learned in the learning phase and your confidence level about your judgment.

[Uncertain competitive outcome feedback]: Only your own task performance will be given to you after the competition ends.

[competition-outcome feedback]：The competition outcomes will be given to you after the competition ends, indicating whether you win or lose.

After performing the competition task, you will also need to provide self-reported ratings.

**The second stage:**

In the second stage, you need to participate in public goods game with your previous competition partner in the first stage.

In the second stage, you need to complete three rounds of PGG tasks. In each round of the task, you have 100 tokens (equivalent to 10 yuan) of investment capital that can be used for investment, which may bring profits or losses. You can make decisions based on your own situation.

**Instruction for control condition**

Hello, welcome to the experiment.

Let me briefly introduce the basic situation of the experiment. The current experiment consists of two stages.

**The first stage:**

The first stage is a memory task in which you need to perform a memory task. There will be anotehr person who will also perform the task. Both of you perform the task independently. The memory task is divided into three phases: learning, distractor and recognition phase. In the learning stage, you need to learn a series of word phrases and try to remember as many as possible. After learning, you need to perform some simple arithmetic operations. Then, in the recognition stage, you need to judge whether some words were learned in the learning phase and your confidence level about your judgment.

Only your own task performance will be given to you after the memory task ends.

After performing the competition task, you will also need to provide self-reported ratings.

The second stage:

In the second stage, you need to participate in public goods game with your previous player in the first stage.

In the second stage, you need to complete three rounds of PGG tasks. In each round of the task, you have 100 tokens (equivalent to 10 yuan) of investment capital that can be used for investment, which may bring profits or losses. You can make decisions based on your own situation.
